# Incorporating Social Determinants of Health in Electronic Health Records: Qualitative Study of Current Practices Among Top Vendors

**DOI:** 10.2196/13849

**Published:** 2019-06-07

**Authors:** Maysoun Freij, Prashila Dullabh, Sarah Lewis, Scott R Smith, Lauren Hovey, Rina Dhopeshwarkar

**Affiliations:** 1 NORC at the University of Chicago Bethesda, MD United States; 2 Centers for Medicare and Medicaid Innovation Baltimore, MD United States; 3 Office of the Assistant Secretary for Planning and Evaluation Washington, DC United States

**Keywords:** electronic health records, social determinants of health

## Abstract

**Background:**

Social determinants of health (SDH) are increasingly seen as important to understanding patient health and identifying appropriate interventions to improve health outcomes in what is a complex interplay between health system-, community-, and individual-level factors.

**Objective:**

The objective of the paper was to investigate the development of electronic health record (EHR) software products that allow health care providers to identify and address patients’ SDH in health care settings.

**Methods:**

We conducted interviews with six EHR vendors with large market shares in both ambulatory and inpatient settings. We conducted thematic analysis of the interviews to (1) identify their motivations to develop such software products, (2) describe their products and uses, and (3) identify facilitators and challenges to collection and use of SDH data—through their products or otherwise—either at the point of care or in population health interventions.

**Results:**

Our findings indicate that vendor systems and their functionalities are influenced by client demand and initiative, federal initiatives, and the vendors’ strategic vision about opportunities in the health care system. Among the small sample of vendors with large market shares, SDH is a new area for growth, and the vendors range in the number and sophistication of their SDH-related products. To enable better data analytics, population health management, and interoperability of SDH data, vendors recognized the need for more standardization of SDH performance measures across various federal and state programs, better mapping of SDH measures to multiple types of codes, and development of more codes for all SDH measures of interest.

**Conclusions:**

Vendors indicate they are actively developing products to facilitate the collection and use of SDH data for their clients and are seeking solutions to data standardization and interoperability challenges through internal product decisions and collaboration with policymakers. Due to a lack of policy standards around SDH data, product-specific decisions may end up being de facto policies given the market shares of particular vendors. However, commercial vendors appear ready to collaboratively discuss policy solutions such as standards or guidelines with each other, health care systems, and government agencies in order to further promote integration of SDH data into the standard of care for all health systems.

## Introduction

**Figure 1:**
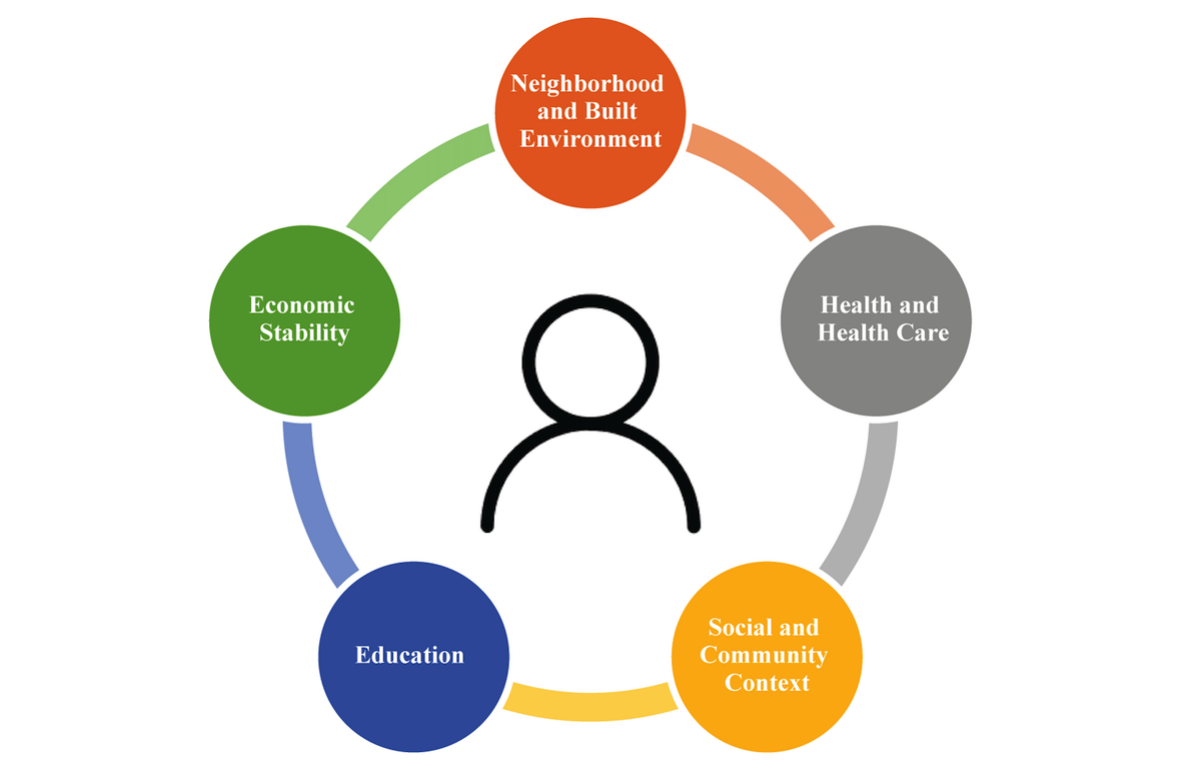
Social determinants of health. Adapted from: Healthy People 2020: Social Determinants of Health [3].

Health care reform initiatives over the past decade have incentivized value-based care payment models and the adoption and development of electronic health records (EHRs) [1,2]. Emphasis on value over volume has drawn attention to the importance of social determinants of health (SDH) in potentially affecting health outcomes. SDH include a wide range of social, economic, and environmental factors that contribute to the health of individuals (Figure 1) [3].

A 2014 report by the National Academies of Medicine (NAM) argued that the integration of SDH into EHRs would better enable health providers to address health inequities and support research into how social and environmental factors influence health [4]. Federal initiatives have spurred SDH data collection through EHRs, including the Comprehensive Primary Care Plus (CPC+) model and Medicare Accountable Care Organizations (ACOs) and Accountable Health Communities (AHCs) [5]. The Centers for Medicare & Medicaid Services (CMS) 2016 Medicaid Managed Care rule has encouraged states to include more community-based, nonclinical services that may address SDH [6,7]. At the local level, health care providers, health departments, universities, legal aid, and social service organizations are developing health improvement interventions that rely on the collection and use of SDH data [8].

Numerous screening tools and approaches have been developed to screen and address SDH [9,10]. Three widely recognized SDH screening tools in the United States are (1) the NAM (2014) set of social and behavioral measures [11]; (2) the National Association of Community Health Center (NACHC) Protocol for Responding to and Assessing Patients’ Assets, Risks, and Experiences (PRAPARE) tool [12]; and (3) the Center for Medicare and Medicaid Innovation’s Accountable Health Communities tool [13]. These tools vary in terms of the overall number of domains or questions, and health care organizations may choose to include additional SDH domains or measures to meet all the needs of their patients. A recent study of six health systems found they all included domains in their SDH screening tools that are not among NAM’s recommended domains, including housing, food insecurity, and transportation [14]. By adapting screening tool questions and domains, organizations have effectively created many different SDH screening tools. Lack of standardization for incorporating data from various screening tools and measures has limited the usefulness of the data within and across EHR systems [15].

With expanded government interest in value-based care (VBC) and quality, health information technology companies that serve as EHR vendors have had both indirect and direct roles in working with policymakers and health care systems. Their indirect role in policymaking has occurred through partnerships with the federal government, health care systems, and other technology companies [16,17]. In forging these relationships, policymakers have directly contributed to the evolution of EHR vendors’ interest in actively engaging in population health as opposed to only developing medical record-keeping products [18,19].

Further, vendors are increasingly incorporating SDH into their EHRs as a way to help their clients respond to the anticipated quality demands of value-based purchasing [20]. Some have dubbed this as a shift from EHRs to comprehensive health records [21]. While health care systems may influence the development of EHR features, there are concerns that the large market shares of relatively few EHR vendors may make vendors less responsive to designing EHRs to meet patients’ and clinicians’ needs, particularly while controlling costs and promoting interoperability [22].

Because of their unique position at the nexus of health systems and health policies and the significant impact their organizational decisions have on EHR-based data capture and clinical practice, we conducted key informant interviews with top EHR vendors focused on SDH. This paper describes vendor perspectives on current challenges and promising opportunities to improve the capture and usability of SDH data in EHRs.

## Methods

We began with a scan of PubMed for peer-reviewed literature and grey literature involving EHRs, SDH, and/or health disparities. Results were limited to articles published in English between January 2012 and June 2018. Through a preliminary review of over 250 articles, we identified 52 for in-depth review and thematic analysis of current practices for collecting and using SDH data through EHRs, uses of SDH data in EHRs for clinical care, and promising opportunities for improving such data collection.

Building on this information, we conducted key informant interviews with research and product development staff at EHR vendor companies to learn more about their current activities related to the integration of SDH in EHRs. To draw a purposive sample, we identified 10 vendors with the largest market shares in hospital and ambulatory settings (a total of 17 vendors) and selected the three vendors that held the largest shares in both settings. We then included the three other vendors among the five vendors with the largest shares in either inpatient or ambulatory settings, for a total of nine vendors [23,24]. Through email solicitation, we gained participation from six vendors but were unable to reach appropriate staff for three vendors during the study period. One to three representatives for each vendor joined the phone interviews in March and April 2018. The vendor and participant names have been kept confidential. The interviews were 60 minutes in length and were audio-recorded and transcribed for the purposes of analysis. We explored motivators, successes and facilitators, challenges and barriers, and lessons learned from SDH product development and solicited feedback for policymakers to consider that would improve the collection and use of SDH data for patient care. This study was reviewed and approved by the University of Chicago’s Institutional Review Board.

We conducted a thematic analysis of interview transcripts using NVivo software (QSR International Pty Ltd). To conduct this analysis, we developed a code book based upon topics discussed during the interviews and also a conceptual model that emerged from the interviews. In the conceptual model, vendors’ clients (ie, health care systems or providers) have their own interests and preferences in relation to the policy environment, needs of their patients, resources in their community, and their own models of health care. Health care system clients provide sites for implementation and testing of SDH tools and are often part of the development of the vendors’ SDH products themselves. Our analysis explored the intersection of health policy and health systems in vendor perspectives on SDH product development.

The code book included definitions of individual codes related to policy demands, client demands, vendor’s motivators’ and experiences, SDH data sources and products, research and development of SDH products, implementation experiences, and vendor requests in terms of policies or strategies to facilitate the collection and use of SDH data through EHRs. A senior researcher developed the codebook and trained three research analysts to each code two to three interviews that they had observed and transcribed. The team met to review and discuss the coding process. Testing of intercoder reliability involved multiple staff coding samples of the same text using an initial codebook. We revised the codebook and refined code definitions as needed to assure consistency across staff coding styles. The senior staff also reviewed coded transcripts to assure accuracy and consistency in coded material. Once transcripts were coded, the authors integrated and interpreted findings across codes to understand current practices in the development of SDH-related products in EHRs and the challenges and opportunities for using these products to address patients’ nonmedical needs in health care settings.

## Results

### Motivators of Social Determinants of Health Product Development

All vendors in our sample stressed the importance of meeting their clients’ needs and demands. One of the main drivers of their clients’ interests in collecting and using SDH in the course of health care delivery is the expansion of VBC programs. Vendors cited Patient-Centered Medical Homes, CPC+, and ACOs as motivating their clients to ask for SDH products within their EHRs. Two vendors noticed the most demand came from federally qualified health centers (FQHCs) or community health centers, whereas another observed more widespread interest from academic medical centers, integrated delivery systems, and pediatric and/or specialty groups, stating, “there is interest, not only in utilizing [SDH] from a workflow standpoint, but also making sure that [SDH] becomes an integral part of the patient’s story over different settings, so that it’s becoming more [of a] norm as part of the handoff between care settings.”

Additionally, four vendors identified the Promoting Interoperability (formerly Meaningful Use) incentives for EHR use and Office of the National Coordinator for Health Information Technology (ONC) health IT certification requirements as main drivers for the integration of SDH in EHRs. As a result, all providers using certified EHRs are collecting some SDH data (ie, race, ethnicity, gender identification, and sexual orientation), although they may not necessarily view it or act upon it as such. One vendor explained, “with Meaningful Use, every practice has access to EHRs and there is an immense amount of data that is available [that] has not widely been used for outcomes data research,” such as research or interventions on SDH.

### Types of Social Determinants of Health Products and Their Use Cases

**Figure 2:**
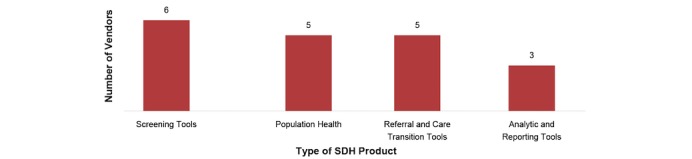
Types of available social determinants of health (SDH) tools and products among sample of vendors (n=6).

Vendors in the study sample varied in their level of investment and development of SDH products. Our findings affirm that the types of SDH products created and used by vendors varies greatly based on their client needs and input and their own strategic planning. In general, vendors have or are in the process of incorporating SDH data in screening tools, population health management tools, tools to improve referral management, and analytic tools (Figure 2).

### Screening Tools Are the Most Common Type of Social Determinants of Health Product

All vendors mentioned the use of screening tools as a part of their platform to collect SDH data. Among the types of screening tools, most vendors described using a configuration of the NACHC’s PRAPARE tool due to their clients’ current demands and its use of structured data and distinct outcomes. PRAPARE EHR templates exist on most top vendor platforms, and the tool is also free as part of a publicly available toolkit [12]. Beyond enabling clients to use the PRAPARE tool or collect whatever other SDH data they choose through their EHRs, one vendor has developed a fully integrated screening product that includes eight NAM recommended measures and two from PRAPARE. Another is working through the intellectual property rights to fully integrate PRAPARE into applications to make it more usable for all of its clients and not just community health centers. Most vendors also described the use of standardized tools to capture data on behavioral health—a common SDH domain—including the Columbia-Suicide Severity Rating Scale [25] and 9-Item Patient Health Questionnaire [26].

All vendors described offering clients multiple or customizable screening tools to focus on fewer or additional measures as needed. As one vendor explained, “Our overarching strategy is to collect SDH data at the individual level in a structured way that is flexible for clients.” Another vendor described the multiplicity of screening tools its clients use and the back-and-forth dynamic with clients that ultimately leads to the development and tailoring of tools:

A number of organizations were using [our social history] form that has been there for a long time. They were creating their own forms to be able to collect this data in a variety of different ways. In some cases they were using other tools, such as the PRAPARE tool, that a number in our group liked and adopted, and it made sense... And so, in some cases it really is...customers being innovative and using different tools and giving us feedback that is determining the best way for us to standardize this on a go-forward basis. We certainly never want to restrict customers from doing what they think they need to be successful or to be innovative.

Variation in screening tools was attributed to the variable demands of particular patient populations (ie, pediatrics) and to the lack of common screening requirements across different federal or state programs. One vendor explained the challenges of developing screening tools that account for federal and state requirements and clients’ preferences and integrate into providers’ workflows:

Mostly what I’ve seen is each state has a different set of requirements in terms of content, questionnaires, screening tools.... There is variation in requirements from state to state or even in a state depending on the practice size or if they are an FQHC [Federally Qualified Health Center]... [Also] some things [may be] a standard [measure] when it comes to a federal requirement but [how] some [measures] are [collected may be] more specific to [a client’s] workflow. In which case we have to make [the measures] go into different sections [of the EHR rather than be in one form that matches the federal requirements]. [The requirements] break the flow sometimes. The customers just want ease of documentation so the challenge is how we can bring everything together into one place. Some being structured data that is standard and some being nonstandard customer specific data.

While screening tools are a common way of capturing SDH data, vendors also described a number of places where SDH data could be collected or found. These include EHR-specific data sets or forms, problem tables, free-text fields located in various places (eg, social history section, clinical notes and assessments section, details section of structured screening tools), the demographic section of the patient’s health record, and the patient portal.

### Population Health Management is a Common Use Case for Social Determinants of Health Data

Three vendors described the development of proprietary population health management tools capable of using algorithms, extracting data, and/or researching community-level patient needs. Although there is not widespread use of SDH data in population health initiatives, one vendor expected that they could be used for diabetes management and food security or medication adherence and utilities. Another described analysis of opioid use, pain tolerance, and pain medication abuse mapped to SDH in areas of opioid addiction. One vendor also described a common request from clients to use secondary survey data to identify “hot spots” or areas of high social need in the communities they serve. It uses data from the CDC Social Vulnerability Index to improve providers’ understanding of community-level social health needs [27]. All vendors recognized growing demand from clients to, as one put it, “move the needle in population health.”

### For Most Vendors, the Use of Referral Products is Still in Early Development or Newly Integrated Into Their Platform

For the five vendors with products capable of making referrals for community services, the common methods are (1) the use of a third-party tool like Aunt Bertha [28], (2) using an EHR-integrated tool like order forms, or (3) using a proprietary tool that allows information exchange among health care systems and outside service providers. These tools are capable of improving care transitions, finding community resources available within a specified radius of a patient’s home address, providing a list of requests or interventions that have been recommended for a patient or assigning a patient to a certain referral program, and providing direct messaging between clinical providers and community-based social service providers for a warm handoff and coordination of complex cases. One vendor describes options that clients have in creating and using referral tools:

One tool that [we] developed is a search tool that finds community resources given the SDH factors that are at the highest risk. For example, using the patient’s home address, we can look within say a 5-mile radius and show all of the transportation services or all of the food pantries. In order to do so our customers can build a list [themselves] or use a third-party vendor that can compile a list that helps them manage the rapidly changing community landscape. Relying on a [third-party] vendor in this space is a strategy that makes sense.

Further, the vendor has created a portal so that the health system and the community service provider can communicate about shared clients. One interviewee explained:

The portal was really to close that loop from a community referral perspective so that they could be on the same care team, they could share parts of the record as appropriate, and they could even contribute feedback by way of notes or simple assessments to really round out the whole picture of someone’s care.

The vendor views such tools as a way of connecting to community-based service providers that historically have not used EHR products but that are integral to addressing the whole health of a patient.

Other vendors also want to close the feedback loop with information on whether patients followed through or benefited from the referral and to have that information reflected in the EHR. Typically, this is done by someone on the clinical care team documenting that the referral has been fulfilled. However, as one vendor observed, among community health clinics, referrals are often made to a service offered within a clinic’s facility or by phone to known community-based service providers; as such, these interactions are not commonly documented in the EHR. Vendors also recognized that there is a lack of consistency in how referrals are documented or managed across EHR systems due to variations in standards implementation, proprietary designs, and also challenges with simply making electronic referrals from health systems to community service providers.

### Vendors Varied in Their Ability to Provide Data Analytics and Reporting

Similar to the use of referral products and capabilities, vendors are still in the early stages of developing mechanisms for analytics and reporting related to SDH. Three vendors interviewed reported using SDH data from the EHRs for risk stratification and outcome assessment. One mentioned the specific use of SDH for reporting to Medicaid for VBC incentives. Another described the use of analytics and reports for following a patient’s progression but was unsure if there is a specific mechanism for reporting SDH. Specifically, the vendor noted concerns with maintaining flexibility in screening tools available to clients and mapping those tools to the same field for analysis. One vendor described strategic development efforts to allow SDH to be included in existing report functions with the goal of better enabling the identification of gaps in care and population management.

In terms of assessing health outcomes, vendors report that measuring both short-term outcomes, such as the completion of the referral, and long-term outcomes, such as changes in costs, utilization, and health outcomes, are difficult both technically and due to challenges addressing SDHs. One vendor has observed clients defining impacts in terms of quality metrics such as reducing readmission rates or reducing emergency department use; it reported that one client assessed outcomes from the person’s perspective of their wellness.

To develop better analytic tools, one vendor has developed a proprietary value set which it is analyzing for the development of risk algorithms that incorporate SDH. It has found that SDH indicators are highly concentrated among a third of the clients or that 30% of clients have collected 90% of the SDH that have been found in the data set. Further, it reports that 90% of what is being collected is only for 13 types of SDH measures, namely separation or divorce, death in the family, unemployment, problems living alone, addiction in family, and caregiver roles; less common are issues like homelessness or child abuse.

### Coding Standards and Interoperability

Data standards are codes for the capture and exchange of electronic health data that govern and ease their integration with other data sets for analysis and use. Specifically, vendors report the use of *International Statistical Classification of Diseases and Related Health Problems, 10th Revision* (ICD-10) and accompanying Z-codes, Logical Observation Identifiers Names and Codes (LOINC) and Systematized Nomenclature of Medicine (SNOMED), and current procedural terminology (CPT) codes, which are necessary for the standardized coding of multiple aspects of the patient record [29-31]. To screen for SDH, there are LOINC and SNOMED codes that cover the same SDH domains; to assess or diagnose SDH, there are SNOMED and ICD-10 codes that cover the same diagnosis; to document an intervention on an SDH (ie, making a referral), there are SNOMED and CPT codes that cover the same procedures [32]. In addition to having multiple terminologies of codes, there are also multiple codes within the same SDH domain.

Due to a lack of standardization, vendors described challenges with the multiplicity and ambiguity of coding SDH measures. One vendor explained, “When looking at the ICD-9 codes, there are about 45 codes that can be used for SDH and when you look at cross-walking those there are about 127 codes in SNOMED that link back to a SDH.” Another vendor described challenges that emerge from the absence of standard terminology. For example, since LOINC and SNOMED do not provide codes for transportation assistance, practices may use a dummy CPT code to track it.

Vendors reported that even with well-known tools like PRAPARE, vendors must sometimes make idiosyncratic coding decisions. In general, the PRAPARE tool has very specific questions and answers—for example, a click list of options for level of education that can link to LOINC terms for each of the responses. Where the ambiguity arises is mapping questions like, “What is the highest level of school you’ve finished?” Although the LOINC and SNOMED answer options might be the same, the vendor would not feel comfortable making the decision to code to one terminology over the other. From the vendor perspective, ideally PRAPARE would be hard coded to a single standard to ensure consistency and interoperability.

Further, not all SDH information can be coded, and free-text fields are frequently used. In spite of the tens of thousands of codes among ICD, LOINC, and SNOMED, some vendors commented that a lot of information that is collected cannot be characterized by a given code and falls into free text. One vendor explained:

In an ideal world all of this [SDH] information would be collected in a codified way, and there would be a table where they can see all of this information. However, in the world today all of the information can be variable in terms of where and how it is collected. It sometimes comes up in the problem table, but we have not begun to even look at the free-text physician notes section, where they anticipate even more information may be collected.

Three vendors reported that some depression surveys are challenging to analyze because they combine yes/no questions with free-text fields intended to capture more detailed information about the patient. Clients appreciate being able to capture these explanations from patients via the free text, in spite of the challenges with codifying them.

In terms of interoperability, lack of standards in both what SDH data is collected and how it is coded also makes its exchange among health care providers difficult. While vendors can use the Consolidated Clinical Document Architecture (C-CDA) to make electronic referrals to community service providers and support system-to-system exchange, one vendor explained that the C-CDA does not codify specific SDH data elements.

Another vendor reported working on a project with some Regional Health Information Exchange Organizations (RHIOs) interested in receiving SDH data. They are starting with race and ethnicity with the intent of sending additional information as the project develops and anticipate other RHIOs will express similar interest.

Finally, vendors described challenges with analysis of SDH data due to lack of standardization. One vendor spoke of the need to standardize or structure SDH data while preserving client flexibility in its collection. An interviewee explained:

If the data is more structured, the analysis is easier. If we have to scale to many clients, with many different screening tools, our job is not to force into one screening tool, but is to normalize the results of the screening tools, so we can map food insecurity tools A and B to the same field that can then be used for analysis. As an IT vendor that kind of data structure is very important.

**Figure 3:**
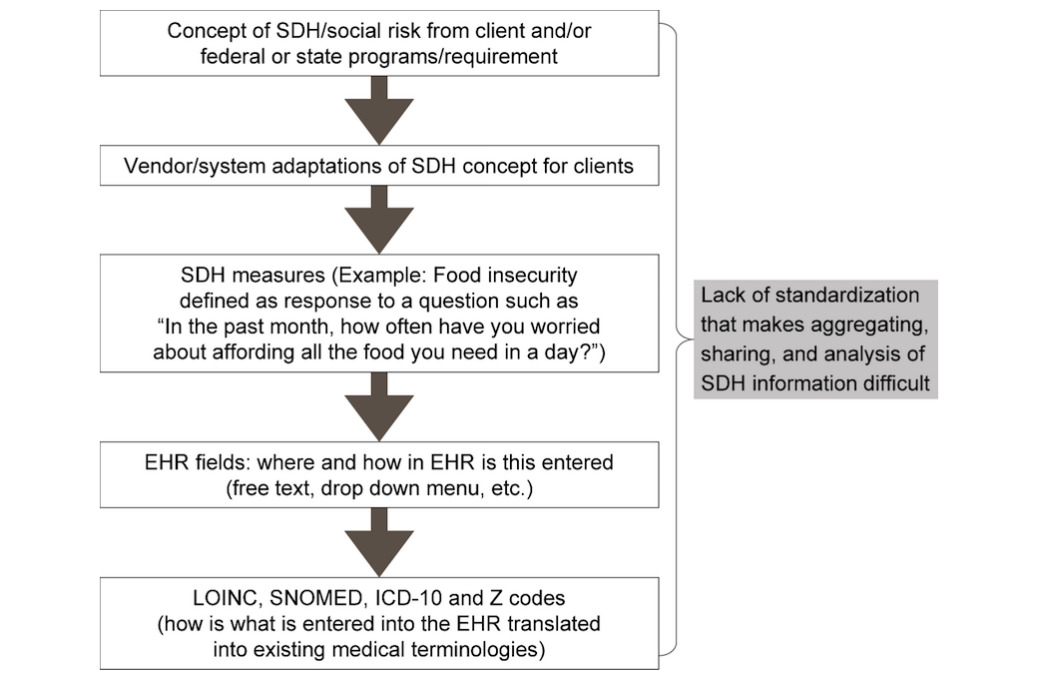
Systemic variability leading to lack of standardization and usability of social determinants of health data. SDH: social determinants of health; EHR: electronic health record; LOINC: Logical Observation Identifiers Names and Codes; SNOMED: Systematized Nomenclature of Medicine; ICD-10: International Statistical Classification of Diseases and Related Health Problems, 10th Revision.

Although clearly the benefit of standardization was viewed from the perspective of the potential benefit to the vendor itself, it is understood that generally better standardization would allow health systems to better analyze and interpret SDH data in clinical decision-making. Figure 3 depicts the chain reaction of variability that leads to the lack of standardization and its limits on the use of SDH data in patient care and population health planning.

### Vendor Recommendations on Standardization

Vendors in this sample indicated that in the absence of national standards, clients are getting “pretty creative” in the collection and use of SDH data. Vendors showed support for discussions among vendors, standards bodies, and government organizations to reduce ambiguity in the code sets, as well as to ensure all voices are heard. Ultimately, several emphasized that vendors must follow the recommendations that public agencies outline and sought direction on standardized tools to collect SDH data, standards for SDH data coding and interoperability, and incentives for SDH data collection and use.

### Standardized Tools to Collect Social Determinants of Health Data

Vendors generally agreed that having standardized definitions of SDH across all government programs would improve the field from a research and analytics perspective. It would also help vendors build tools that are more interoperable. Specifically, if different federal programs can agree on a set of measures, it would facilitate more standardization. For example, one individual commented that, “The PRAPARE tool is great, but the private sector does not seem to be open to it, and it is not an exact match to some of the other national programs already, so there is some disconnect there” that leads to the implementation of differing SDH tools across health systems.

### Standards for Social Determinants of Health Data Coding and Interoperability

Vendors encouraged the use of standard terminology to enable interoperable exchange of SDH-related data. In some cases, more than one standard is assigned to a particular data element. Vendors would appreciate guidance on the preferred standard to be used for a minimum set of data elements. However, they also caution that not all elements can be codified, and how a specific tool is implemented in the EHR should be at the client’s discretion. In particular, this relates to making determinations about the tools that are most useful to their practices, with the recognition that the data they capture must roll up to meet federal reporting standards.

Vendors are involved in discussions and workgroups related to SDH standards that promote data capture and interoperability (Textbox 1). Some participated in national standards development organization activities like the Health Level-7 International C-CDA standards workgroup. Some were involved with nongovernmental initiatives such as one led by the Social Interventions Research & Evaluation Network (SIREN) to improve interoperability of SDH data in EHRs [33]. Finally, vendors continue to engage in industry efforts focused on health information exchange. One vendor reported participation in an industry-wide interoperability initiative called Carequality that grew out of the Sequoia Project [34].

**Two vendors’ views on their role in creating coding standards.**

Yes, we have a role to play [in developing standards for coding social determinants of health data], but we also want to be cognizant of the optics and want other vendors to participate. We don’t want to be perceived as commandeering the narrative.

It’s hard as an [information technology] company to push a standard, because others may perceive it as bias. When an open standard for social determinants is pushed from a national group it is better and that’s something we support.

### Incentives for Social Determinants of Health Data Collection and Use

From the demand side, clients drive demand, investment, and more development, as do policies, including incentives and VBC programs. However, vendors wonder whether the incentives will be fair and whether SDH collection is a fad versus a priority with longevity. One vendor posed the question of whether SDH will come to be as large a movement as quality improvement was for health care.

## Discussion

### Principal Findings

**Figure 4:**
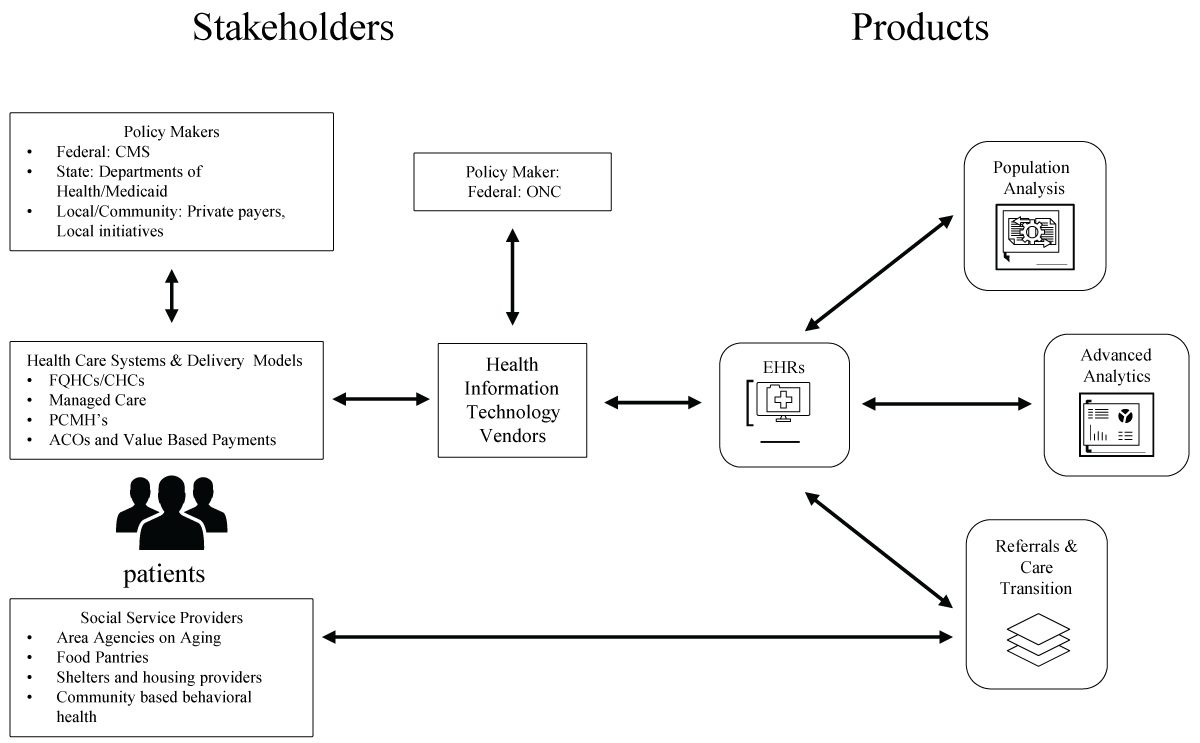
Stakeholders that inform vendors’ social determinants of health–related products in electronic health records. CMS: Centers for Medicare and Medicaid Services; ONC: Office of the National Coordinator for Health Information Technology; FQHC: Federally Qualified Health Center; CHC: community health center; PCMH: patient-centered medical home; ACO: Accountable Care Organization; EHR: electronic health record.

Vendor systems and their functionalities are the result of the multiple, interrelated forces of federal policy and regulation, client demand, and the vendors’ own strategic vision for opportunities in the health care system. Through interviews with vendors, we explored the roles of client demand and federal policies related to SDH capture and use. We also explored issues related to use of standards and interoperable information sharing, use cases for SDH to improve clinical care and processes, and potential avenues for growth in use of SDH data. In doing so, we see the influence of numerous stakeholders—federal, state, and local policy makers; health systems; social services systems; health information technology vendors; and patients—on the development of SDH-related products in EHRs (Figure 4). Health information technology companies that serve as EHR vendors must adhere to federal policies set out by ONC, and health care systems and the delivery models they use must adhere to federal policies set out by CMS (among others) as well as state and local health-related policies. Both types of stakeholders may also have some influence on such policies as well. The SDH-related products that vendors make to enable population analysis, advanced analytics, and referrals and care transitions seek to better integrate care delivered in health care settings with social services outside of those settings, thereby addressing patients’ nonmedical needs. Yet many interests, policies, and products need to align in order for this to happen.

In this study, we found that even among vendors with large market shares in both ambulatory and inpatient settings, SDH is a new area for investment, and there is room for growth in terms of product development and analytic capacity. While all vendors interviewed use or have enabled some SDH data collection screening instruments or measures in their EHR platform, they vary in terms of capacity to track referrals and analyze data. Vendors activities also ranged from simply seeking to help clients meet regulatory obligations to those engaged in research to develop products that will help clients better target and address needs, including those related to SDH, of their patients.

Vendors identified a number of challenges primarily with analyzing SDH data and sharing them among health systems. This includes challenges with multiple overlapping but distinct performance metrics and indicators across various federal and state programs, lack of agreement on mapping SDH measures to codes, and lack of codes for all measures. Finally, there is a general problem with interoperability among different health care systems that makes sharing and using SDH data difficult. Vendors appear to have taken a role in resolving these challenges through participation in policy development, standardizing bodies, and vendor-specific solutions and decisions. With the lack of policy regulations around SDH data, product-specific decisions may end up being de facto policies given the market share of particular vendors. However, vendors appear ready for formal policymaking discussions to seek solutions that may further promote the integration of SDH data into mainstream health care delivery.

### Limitations

The findings from this study are based on a purposive, qualitative sample with a small number of vendors. They are not intended to represent the state of the EHR field at large but rather to help identify trends in the development and use of SDH screening tools and data among vendors with considerable stake in this area given their market shares in inpatient and outpatient settings. The study was also limited to vendors we could reach during a limited study period. With more time, we would have sought more input from representatives working on this increasingly commercialized component of health care systems.

### Conclusions

In order to advance the collection and use of SDH data in health care settings through EHRs, the findings from this study suggest at least three next steps:

- Identify core SDH measures where standard development is still needed. For example, since LOINC and SNOMED do not provide codes for transportation assistance, additional code development may be needed.
- Provide guidance on preferred terminology standards for some SDH measures. For example, since education and bereavements have several codes that can be used, providing guidance on preferred terminology would eliminate vendors and health care organizations making idiosyncratic coding choices.
- Identify standards for a subset of SDH measures that health systems can routinely collect through EHRs. Building upon earlier work by ONC to require certified EHRs to collect SDH measures such as race and ethnicity, initiatives to develop standards around specific SDH domains may help encourage their widespread use in EHRs. SIREN and its Gravity Project are a current example of such an effort. This national collaborative seeks to promote interoperable documentation of three priority SDH domains: food security, housing stability and quality, and transportation [35].

This study has shown that in the absence of standardization of SDH screening instruments, measurements, and codification, EHR vendors will provide their clients multiple options and flexible tools to meet their varying needs and interests. We were limited to a small number of vendors that we could reach in a short time frame, but the vendors have large market shares and were consistent in the need to remain adaptable and responsive to client needs and federal and state requirements. They appreciated the potential for standardized SDH data to identify patients with high social need, improve care coordination between health care providers and community service providers, and build further evidence on the connections between SDH and health outcomes through better data analytics and population health management. Vendors and providers seek approaches that balance the use of existing data with the need to collect standardized new data in order to streamline the integration of SDH data in providers’ workflow and create a holistic picture of patients that may ultimately reduce health disparities.

## Abbreviations

ACO: Accountable Care Organization
AHC: Accountable Health Community
C-CDA: Consolidated Clinical Document Architecture
CMS: Centers for Medicare & Medicaid Services
CPC+: Comprehensive Primary Care Plus
CPT: current procedural terminology
EHR: electronic health record
FQHC: federally qualified health centers
ICD-10: International Statistical Classification of Diseases and Related Health Problems, 10th Revision
LOINC: Logical Observation Identifiers Names and Codes
NACHC: National Association of Community Health Center
NAM: National Academies of Medicine
ONC: Office of the National Coordinator for Health Information Technology
PRAPARE: Protocol for Responding to and Assessing Patients’ Assets, Risks, and Experiences
RHIO: Regional Health Information Exchange Organization
SDH: social determinants of health
SIREN: Social Interventions Research & Evaluation Network
SNOMED: Systematized Nomenclature of Medicine
VBC: value-based care

## References

[1] Burwell SM (2015). Setting value-based payment goals—HHS efforts to improve U.S. health care. N Engl J Med.

[2] Azar A (2018). Remarks on Value-Based Transformation and Innovation.

[3] Healthy People 2020: social determinants of health.

[4] Institute of Medicine of the National Academies (2014). Capturing Social And Behavioral Domains In Electronic Health Records: Phase 1.

[5] (2016). Health IT in the Quality Payment Program.

[6] Machledt D Addressing the social determinants of health through Medicaid managed care.

[7] Spencer A, Freda B, Mcginnis T, Gottlieb L (2016). Measuring social determinants of health among Medicaid beneficiaries: early state lessons.

[8] De Milto L, Nakashian M (2016). Using social determinants of health data to improve health care and health: a learning report.

[9] Gottlieb L, Cottrell EK, Park B, Clark KD, Gold R, Fichtenberg C (2018). Advancing social prescribing with implementation science. J Am Board Fam Med.

[10] Social need screening tools comparison table.

[11] Institute of Medicine of the National Academies (2015). Capturing Social And Behavioral Domains And Measures In Electronic Health Records: Phase 2.

[12] (2017). The protocol for responding to and assessing patients' assets, risks, and experiences (PRAPARE).

[13] Smith-Bindman R (2010). Is computed tomography safe?. N Engl J Med.

[14] LaForge K, Gold R, Cottrell E, Bunce AE, Proser M, Hollombe C, Dambrun K, Cohen DJ, Clark KD (2018). How 6 Organizations developed tools and processes for social determinants of health screening in primary care: an overview. J Ambul Care Manage.

[15] Cantor MN, Thorpe L (2018). Integrating data on social determinants of health into electronic health records. Health Aff (Millwood).

[16] (2018). Meaningful Use: public health–EHR vendors collaboration initiative.

[17] (2018). Integrated health model initiative.

[18] Tripathi M (2012). EHR evolution: policy and legislation forces changing the EHR. J AHIMA.

[19] Glaser J (2015). From the electronic health record to the electronic health plan.

[20] Bresnick J (2018). Faulkner guides Epic Systems from EHR to comprehensive health record.

[21] Sullivan T (2017). Epic's rival EHR vendors say they too are making the CHR switch.

[22] Koppel R, Lehmann CU (2015). Implications of an emerging EHR monoculture for hospitals and healthcare systems. J Am Med Inform Assoc.

[23] (2017). Certified Health IT developers and editions reported by hospitals participating in the Medicare EHR Incentive Program.

[24] (2017). Certified Health IT developers and editions reported by ambulatory primary care physicians, medical and surgial specialists, podiatrists, optometrists, dentists, and chiropractors participating in the Medicare EHR Incentive Program.

[25] The Columbia-Suicide Severity Rating Scale (C-SSRS).

[26] Kroenke K, Spitzer RL, Williams JB (2001). The PHQ-9: validity of a brief depression severity measure. J Gen Intern Med.

[27] Social vulnerablity index.

[28] Aunt Bertha.

[29] Factors influencing health status and contact with health services Z00-Z99.

[30] (2018). International Classification of Diseases, Tenth Revision, Clinical Modification (ICD-10-CM).

[31] (2018). Supporting interoperability: terminology, subsets, and other resources from NLM.

[32] Arons A, DeSilvey S, Fichtenberg C, Gottlieb L (2018). Compendium of Medical Terminology Codes for Social Risk Factors.

[33] Arons A, Desilvey S, Fichtenberg C, Gottlieb L (2017). Improving the interoperability of social determinants data in electronic health records: working paper for November 2017 expert panel convening. Social Interventions Research & Evaluation Network Stakeholder Meeting.

[34] The Sequoia Project.

[35] The Gravity Project: a national collaborative to advance interoperable social risk and protective factors documentation.

